# Multimodal Detection of a Minute Uterine Cornual Lesion in Cervical Clear Cell Carcinoma

**DOI:** 10.3390/diagnostics16203357

**Published:** 2026-10-15

**Authors:** Peng Lu, Fei Chen, Lixu Xin, Congsong Dong, Zhenyu Dai

**Affiliations:** Department of Radiology, The Yancheng School of Clinical Medicine of Nanjing Medical University, Yancheng Third People’s Hospital, No. 2 Xindu West Road, Yandu District, Yancheng 224005, China

**Keywords:** cervical clear cell carcinoma, uterine cornu, ^18^F-FDG PET/CT, magnetic resonance imaging, ultrasonography, multimodal imaging

## Abstract

Cervical clear cell carcinoma (CCC) is a rare human papillomavirus (HPV)-independent adenocarcinoma. A 73-year-old woman presented with postmenopausal bleeding. Ultrasonography demonstrated a cervical mass and a 5.0 × 5.0 mm right cornual nodule. On 3.0-T magnetic resonance imaging (MRI), the cornual lesion measured 5.2 × 5.3 mm and, like the cervical tumor, showed high signal intensity on diffusion-weighted imaging (b = 1000 s/mm^2^). ^18^F-fluorodeoxyglucose positron emission tomography/computed tomography (^18^F-FDG PET/CT) demonstrated intense uptake in the cervical mass (30.6 × 29.2 × 41.2 mm; SUV_max_ 10.4) and in the spatially separate right cornual nodule (5.2 × 5.1 mm; SUV_max_ 14.6), without FDG-avid nodal or distant metastatic disease. The concordant identification of the cornual lesion by ultrasonography, MRI, and PET/CT prompted multidisciplinary reassessment and radical hysterectomy to obtain definitive pathological characterization of both lesions. Histopathology demonstrated clear cell carcinoma at both sites; however, because molecular, genomic, or clonality analyses were not performed, their biological relationship remains uncertain. All 30 examined lymph nodes and the surgical margins were negative. The cervical primary was staged as pT1b3 pN0 cM0 (FIGO 2018 stage IB3). The patient received four cycles of postoperative chemotherapy with paclitaxel plus cisplatin. At 14 months after surgery, gynecological examination and pelvic MRI showed no evidence of recurrence. This case highlights the complementary roles of ultrasonography, MRI, and ^18^F-FDG PET/CT in characterizing a minute spatially separate uterine lesion while underscoring the need for cautious interpretation when the relationship between lesions cannot be established.

**Figure 1:**
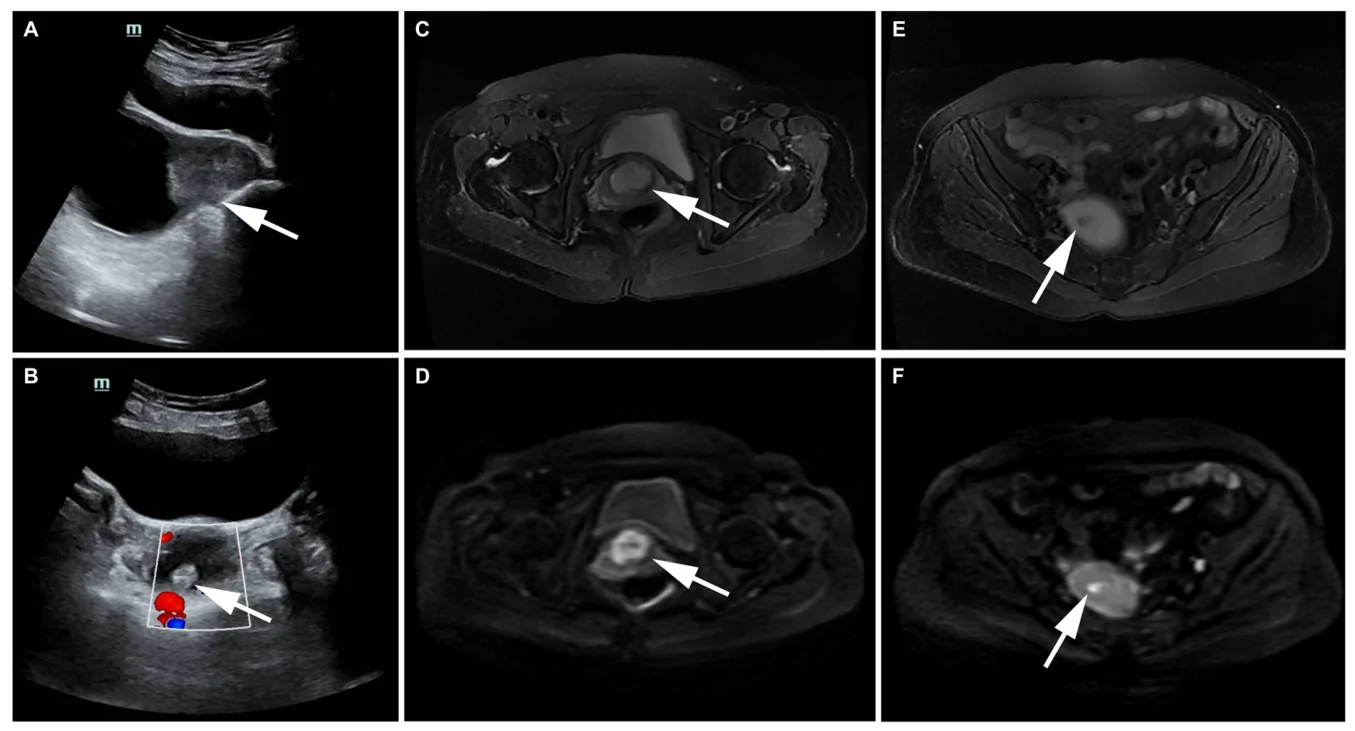
Multimodal ultrasonographic and magnetic resonance imaging findings. A 73-year-old woman presented with postmenopausal vaginal bleeding, and human papillomavirus testing was negative. (**A**) Ultrasonography demonstrates a hypoechoic mass in the uterine cervix (arrowhead). (**B**) Ultrasonography demonstrates a small hypoechoic nodule in the right uterine cornu (arrowhead), measuring approximately 5.0 × 5.0 mm and spatially separate from the cervical mass. (**C**) T2-weighted MRI demonstrates the cervical soft-tissue mass with high signal intensity (arrowhead). (**D**) The cervical mass shows high signal intensity on diffusion-weighted imaging (DWI; b = 1000 s/mm^2^) (arrowhead). (**E**) T2-weighted MRI demonstrates the right cornual lesion (arrowhead), measuring approximately 5.2 × 5.3 mm. (**F**) The right cornual lesion also shows high signal intensity on DWI (b = 1000 s/mm^2^) (arrowhead). MRI was performed on a 3.0-T system. A large amount of fluid is present within the uterine cavity. Cervical CCC is an uncommon HPV-independent endocervical adenocarcinoma [1]. In a clinical series of 18 patients with cervical clear cell adenocarcinoma, vaginal bleeding was the most frequent presenting symptom, and HPV testing was negative in most tested patients [2]. Ultrasonography initially identified the right cornual nodule, while MRI confirmed its spatial separation from the cervical tumor and provided local soft-tissue characterization. The concordant visualization of the nodule on ultrasonography and MRI supported the presence of a true focal lesion rather than an imaging artifact. MRI is complementary to PET/CT in the imaging assessment of cervical malignancy [3].

**Figure 2:**
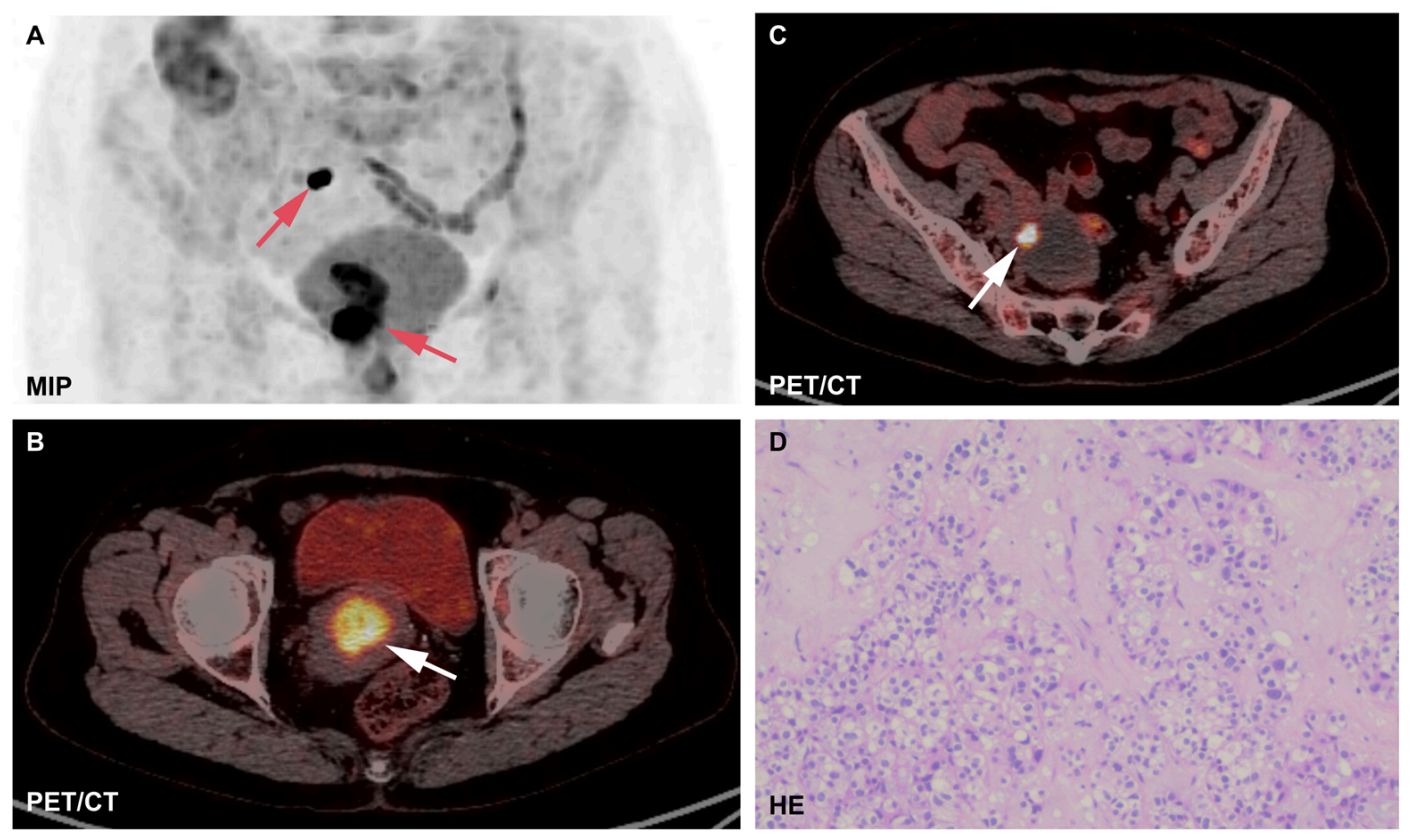
^18^F-FDG PET/CT and histopathological findings. (**A**) Maximum-intensity-projection (MIP) ^18^F-FDG PET image demonstrates abnormal FDG uptake in the pelvis. No FDG-avid nodal or distant metastatic disease was identified on whole-body PET/CT. (**B**) Fused ^18^F-FDG PET/CT demonstrates intense FDG uptake in the cervical mass (arrowhead), measuring approximately 30.6 × 29.2 × 41.2 mm, with an SUV_max_ of 10.4. (**C**) Fused ^18^F-FDG PET/CT demonstrates intense focal FDG uptake in the spatially separate right cornual nodule (arrowhead), measuring approximately 5.2 × 5.1 mm, with an SUV_max_ of 14.6. PET/CT acquisition was performed 60 min after FDG administration using a GE Discovery MI system. PET images were reconstructed with Q. Clear (β = 400) and a reconstructed image spacing of 2 mm. Given the approximately 5 mm size of the cornual lesion, quantitative SUV interpretation is subject to substantial uncertainty from finite spatial resolution and partial-volume effects; SUV_max_ is also influenced by reconstruction and statistical noise. Accordingly, the reported SUV_max_ should be interpreted as a semiquantitative descriptor of focal FDG avidity rather than as an accurate estimate of true tracer concentration or a direct measure of biological aggressiveness [4]. (**D**) Representative hematoxylin and eosin-stained section of the cervical clear cell carcinoma (×400). Ultrasonography initially identified the small right cornual lesion, MRI confirmed its spatial separation and local imaging features, and ^18^F-FDG PET/CT provided complementary metabolic characterization and whole-body staging rather than serving as the sole modality responsible for lesion detection. The patient had initially been considered for radiotherapy for presumed cervical carcinoma. The concordant identification of a second spatially separate cornual lesion introduced uncertainty about disease extent and the biological relationship between the two lesions. Because no nodal or distant metastatic disease was identified, multidisciplinary reassessment favored radical hysterectomy to achieve local disease control and to obtain definitive histopathological characterization of both lesions. PET/CT is an established complementary imaging modality in cervical cancer staging and treatment planning [3,5]. At surgery, a cervical mass involving the cervical wall and a separate cauliflower-like nodule measuring approximately 5 mm near the right uterine cornu were identified, with no macroscopic continuity between them. The absence of macroscopic continuity does not exclude microscopic spread. Histopathological examination demonstrated cervical clear cell carcinoma with invasion of more than two-thirds of the cervical fibromuscular wall. No lymphovascular or perineural invasion was identified. Immunohistochemistry of the cervical tumor showed ER (+), PR (−), p16 (+), Ki-67 labeling index approximately 30%, p40 (−), CEA (−), CD31 (−), D2-40 (−), wild-type p53 expression, Napsin A (+), and HNF1β (+). The spatially separate right cornual nodule also demonstrated clear cell carcinoma with invasion of less than half of the myometrial thickness and no lymphovascular or perineural invasion; Napsin A and HNF1β were positive, and no broader immunohistochemical panel was performed on the cornual lesion. All 30 examined lymph nodes were negative for metastasis, including 14 left pelvic, 5 right pelvic, 6 right obturator, 1 left common iliac, 2 right common iliac, and 2 para-aortic lymph nodes. Surgical margins were negative, with no carcinoma identified at the parametrial or vaginal wall margins. The cervical primary was staged as pT1b3 pN0 cM0 (FIGO 2018 stage IB3). This stage refers to the cervical primary; the right cornual lesion is described separately because its biological relationship to the cervical tumor could not be definitively established. Uterine corpus/endometrial involvement has previously been described in cervical clear cell carcinoma [2]. Napsin A supports clear cell differentiation, whereas HNF1β is less specific in the differential diagnosis between clear cell and mesonephric carcinomas [6]. The differential interpretation of the cornual lesion includes spread from the cervical primary, multifocal disease, or a synchronous uterine clear cell carcinoma. Although both lesions were diagnosed as clear cell carcinoma and shared Napsin A/HNF1β positivity, the available pathological findings cannot establish a common site of origin or clonal relationship. Because molecular, genomic, or clonality analyses were not performed, the biological relationship remains unresolved; therefore, the cornual lesion should not be regarded as a confirmed metastasis. Following surgery, adjuvant treatment options were reassessed. Pelvic radiotherapy was discussed, but the patient declined radiotherapy because of concerns about radiation-related injury to pelvic organs. After multidisciplinary evaluation, systemic therapy was selected in view of the overall recurrence-risk assessment and concern for occult distant micrometastatic disease. The patient received four cycles of postoperative chemotherapy with paclitaxel plus cisplatin. At 14 months after surgery, gynecological examination and pelvic MRI showed no evidence of recurrent disease. This report has two important limitations: first, the absence of molecular/genomic/clonality analysis prevents definitive determination of the relationship between the two tumors; second, the 14-month follow-up period is relatively short and does not permit conclusions regarding long-term recurrence or survival.

## Data Availability

The data supporting the findings of this report are available from the corresponding author upon reasonable request. The data are not publicly available because of patient privacy considerations.
